# Chinese Visceral Adiposity Index and Depressive Symptoms in Middle-Aged and Elderly Chinese: Dose-Response Correlation and the Effect Mediated by Sleep Time and Life Satisfaction

**DOI:** 10.1155/2023/3428722

**Published:** 2023-08-14

**Authors:** Miyuan Wang, Liang Pan, Hongye Peng, Bin Song, Yan Zeng, Mingtao Qian, Qing Yang, Shanshan Xie, Gang Mai, Hui Wang

**Affiliations:** ^1^School of Public Health, Huazhong University of Science and Technology, Wuhan 430074, China; ^2^Phase 1 Clinical Trial Center, Deyang People's Hospital, Sichuan 618000, China; ^3^Graduate School, Beijing University of Chinese Medicine, Beijing 100029, China; ^4^Department of Nephrology, Deyang People's Hospital, Sichuan 618000, China; ^5^Neonatal Intensive Care Unit, Deyang People's Hospital, Sichuan 618000, China; ^6^Heilongjiang University of Traditional Chinese Medicine First Hospital, Heilongjiang 150040, China; ^7^School of Foreign Languages and Culture, Nanchang University, Jiangxi 330036, China; ^8^Deyang Maternal and Child Health Service Center, Sichuan 618000, China; ^9^Department of Anesthesiology, Fudan University Shanghai Cancer Center, Shanghai 200030, China; ^10^Department of Oncology, Shanghai Medical College, Fudan University, Shanghai 200030, China

## Abstract

Depression is a common psychosomatic disorder in clinical practice and may soon become the largest disease burden globally. Studies have focused on the association between obesity and depression but presented controversial results. This study is aimed at exploring the dose-response correlation between Chinese visceral adiposity index (CVAI) and depressive symptoms and the mediating effect of sleep time and life satisfaction in this relationship. We include 4149 individuals aged ≥45 years from wave 2011 and wave 2015 of China Health and Retirement Longitudinal Study (CHARLS), using restricted cubic spline (RCS) to examine possible nonlinear correlation and serial multiple mediation model to examine the mediating effect of sleep time and life satisfaction. Results indicate that there is a significant negative linear correlation between CVAI and depressive symptoms, and each IQR increment in CVAI is associated with 11% lower risk of depressive symptoms. About 50.00% (indirect effect/total effect) of the significant association between CVAI and depressive symptoms is mediated by sleep time and life satisfaction, with life satisfaction playing a relatively major role. Properly visceral adiposity may be protective against depressive symptoms. It could be feasible to alleviate the depressive symptoms in people with too low visceral adiposity by improving their life satisfaction.

## 1. Introduction

As a common psychosomatic disorder in clinical practice, depression is characterized by continuous and persistent depressed mood, lack of interest, loss of energy, and slowed thinking, with high incidence, high relapse rate, high disability rate, high suicide rate, and low cure rate [1]. A meta-analysis indicates that the population suffering from major depression will increase by 27.6% globally due to the COVID-19 pandemic (“Global prevalence and burden of depressive and anxiety disorders in 204 countries and territories in 2020 due to the COVID-19 pandemic,” 2021). According to the World Health Organization, depression is expected to surpass oncological and cardiovascular diseases as the largest disease burden worldwide by 2030. Studies show that depression is associated with reduced physical and psychological functioning, decreased quality of life, and increased suicidal ideation, which seriously effect the physical and mental health of patients [2, 3]. The risk of depression is higher in middle-aged and elderly population because of ageing, degenerative changes in body functions, and changes of social role. Moreover, it is challenging to diagnose and treat depression due to the combination with various underlying diseases [4].

With the increasing number of obese population, the relationship between obesity and depression is gaining attention. According to studies, homeostatic adjustments (including immunoinflammatory activation, hypothalamic-pituitary-adrenal axis, neuroendocrine regulators of energy metabolism such as leptin and insulin, and microbiome), brain circuitries integrating homeostatic and mood regulatory responses may be common biological mechanisms between obesity and depression [5]. Presently, relevant clinical studies have focused mostly on children and adolescents. A Sweden cohort study of 12,507 participants confirmed that obesity was associated with anxiety/depression risks in children and adolescents [6]. A meta-analysis of 69,893 subjects showed that obesity increased the risk of depression in children and adolescents compared to healthy controls (OR = 1.851, 95% CI = 1.410-2.429) [7]. Few studies have looked at the middle-aged and elderly population, and the findings of these studies were controversial. Based on the data from the Health and Retirement Study [8], overweight and obese populations were more likely to develop depressive symptoms (HR = 1.13, 95% CI = 1.04‐1.23; HR = 1.09, 95% CI = 1.01‐1.18, respectively) compared to population with normal weight. However, Luo et al. suggested that obese male had lower risk of depressive symptoms compared to those with normal BMI (HR = 0.506, 95% CI = 0.347‐0.736) [9].

BMI and waist circumference (WC) are the most commonly used indicators in current studies on the link between obesity and depression; however, they do not accurately reflect visceral fat content. The visceral adiposity index (CVAI) is a novel body fat index proposed by Xia et al. and is calculated using age, BMI, WC, triglyceride (TG), and high-density lipoprotein cholesterol (HDL-C), being considered a reliable index for evaluating visceral fat in Chinese population [10]. Previous studies suggest that CVAI has higher disease predictive efficacy compared to BMI, WC, or body shape index [11]. Multiple studies have shown that life satisfaction and sleep disorder have a significant impact on middle-aged and elderly people's health and life quality, and both have a high correlation with depression and obesity [12–15].

Based on the China Health and Retirement Longitudinal Study (CHARLS) database in a prospective cohort, this study is aimed at investigating the efficacy of CVAI in predicting new-onset depression and the dose-response relationship between them, as well as the mediating effect of sleep time and life satisfaction in the relationship between CVAI and depressive symptoms, so as to better understand the correlation between obesity and depressive symptoms in middle-aged and elderly people.

## 2. Material and Methods

### 2.1. Study Population

Study data were collected from CHARLS, approved by Peking University Ethical Review Committee (IRB00001052–11,015), and a freely accessible database at http://charls.pku.edu.cn/en. CHARLS integrated an extensive amount of data from nationwide participants including basic information, family structure, physical condition, health care and insurance, work and pension, income and expenditure, housing, and laboratory examination [16]. Participants of 45 years of age or older from 450 villages/communities in 28 provinces (autonomous regions and municipalities) were surveyed. And 12,241 households with 21,097 participants were included in CHARLS by 2015 in total. Written informed consent was signed by all participants. It was a reliable source for researching the health conditions and possible risk factors for the middle-aged and elderly people.

Samples of fasting venous blood were taken by trained staff and then tested on-site or preserved and transferred to the central lab for further laboratory testing. Enzyme colorimetric assay was employed to assess glucose, total cholesterol (TC), TG, low-density lipoprotein cholesterol (LDL-C), and HDL-C.

This study was conceived as a retrospective longitudinal cohort study based on data from CHARLS. The primary objective was to investigate the relationship between the baseline status of the CVAI and the incidence of new-onset depression symptoms. This study selected participants according to the following inclusion criteria: age ≥ 45 years; with no depressive symptoms at baseline; full basic data such as gender, level of education, marital status, and location; full data of fasting blood glucose. A total of 4,149 qualified participants were eventually included.

### 2.2. Measurement

#### 2.2.1. Assessment of VAI

The distribution and function of fat could be indicated by VAI, an empirical-mathematical model combining anthropometric (age, BMI, and WC) and functional (TG and HDL) factors. Specific formulas were proposed to compute the VAI score for the Chinese population [17]:

Chinese male:

(1) $$\begin{array}{c} \text{CVAI}=-267.93+0.68\times \text{age}+0.03\times \text{BMI}+4.00\times \text{WC}+22.00\times \log10\left(\text{TG}\right)-16.32\times \text{HDL}‐\text{C}. \end{array}$$

Chinese female:

(2) $$\begin{array}{c} \text{CVAI}=-187.32+1.71\times \text{age}+4.23\times \text{BMI}+1.12\times \text{WC}+39.76\times \log10\left(\text{TG}\right)-11.66\times \text{HDL}‐\text{C}. \end{array}$$

#### 2.2.2. Assessment of Depressive Symptoms

The 10-item CES-D was used for depressive symptom assessment [18]. It provided four options for each item, ranging from 0 to 3 (“rarely or none of the time” to “all of the time”). Eight items measuring depressive affect/somatic symptoms scored ranging from 0 to 3, while two items measuring positive affect scored reversely. CES-D scored ranging from 0 to 30, and a higher score indicated more depressive symptoms.

#### 2.2.3. Assessment of Mediating Variables

The following variables were included in the mediation model:

Sleep time: average sleep duration at night of participants in the previous month that was collected from questionnaire and analyzed as continuous variable.

Life satisfaction: degree of life satisfaction evaluated by participants that was collected from a questionnaire with 5-point Likert scale ranging from 1 to 5, and a higher score indicated less life satisfied.

#### 2.2.4. Assessment of Covariates

The analyses were adjusted for sociodemographic factors, health-related activities, and anthropometric data. Age, gender, education (primary school or below, high school, college or above), location (city/town, village), marital status (nonmarried, married), and diabetes (yes, no) were all included as demographic variables. Variables such as smoking status, drinking status, and sleep time were included as health-related activities. All the data were collected from self-reported questionnaires. Using an Omron HEM-7200 sphygmomanometer, the average systolic blood pressure (SBP) and diastolic blood pressure (DBP) were calculated as the mean of the three measurements and used as anthropometric data.

### 2.3. Statistical Analysis

All statistical analyses were completed with R 4.1.3. Statistical significance was defined as a two-tailed *P* < 0.05. Data were expressed with mean (standard deviation (SD)) or median (interquartile range (IQR)) for continuous variables and percentages for categorical variables. Baseline characteristics and the incidence of depressive symptoms were assessed with the ANOVA, Kruskal-Wallis, or chi-square tests after grouping by CVAI quartile. Odds ratio (OR) with 95% confidence interval (CI) of CVAI for depressive symptoms was calculated with three logistic models, including an unadjusted crude model (model 1); a model adjusted for age, gender, education, location, and marital status (model 2); and a model further adjusted for smoking status, drinking status, sleep time, SBP, and DBP (model 3). Results from models were expressed with ORs and 95% CIs. The ability of the CVAI at baseline to predict the risk of depressive symptoms at follow-up was evaluated using the area under the receiver operating characteristic (ROC) curve (AUC). Interaction analysis was performed to determine the effects of changes in sociodemographic factors and health-related activities on the association between CVAI and depressive symptoms. The restricted cubic splines (R package “rms”) were used to explore the dose-response and potential nonlinear relationships between CVAI and depressive symptoms [19].

The mediating effects of sleep time and life satisfaction on the association between CVAI and depressive symptoms were examined with a serial multiple mediation model (R package “bruceR”). And the statistical significance of the mediation effects was examined with the bootstrap method [20].

## 3. Results

### 3.1. Baseline Characteristics

Characteristics of the study population based on CVAI quartile are shown in Table 1. Among the included 4,149 participants, 932 (22.46%) individuals presented depressive symptoms (CES‐D ≥ 10). We found that higher CVAI levels are associated with higher probability of older, female, live in village, ex-smoker, nondrinking, and diabetes, as well as higher levels of life satisfaction, SBP, DBP, BMI, WC, fasting glucose, TG, and HDL-C.

### 3.2. Dose-Response Relationship between CVAI and Depressive Symptoms

**Figure 1:**
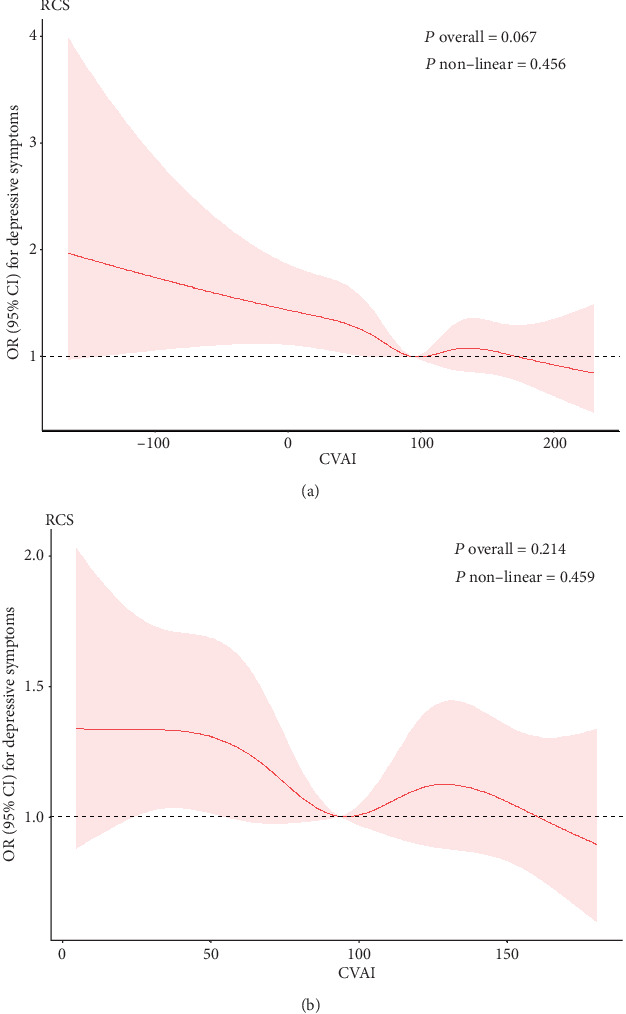
(a) represents the relationship between the CVAI index and the risk of depressive symptoms in restricted cubic spline. (b) represents after excluding the top 2.5% and bottom 2.5% of CVAI values, the relationship between CVAI and depressive symptoms in restricted cubic spline. Age, gender, education level, location, marital status, smoking status, drinking status, sleep time, diabetes, SBP, and DBP were adjusted in the model.

Quartiles of the CVAI index and the correlation between CVAI and the risk of depressive symptoms are shown in Table 2. Compared to the baseline CVAI quartile group (Q1), the Q2 group presented reduced risk of depressive symptoms (OR, 0.76; 95% CI, 0.62-0.95) in model 2. The same result was also observed in the Q2 group (OR, 0.76; 95% CI, 0.61-0.95) after adjusting for age, gender, education level, location, marital status, smoking status, drinking status, sleep time, diabetes history, SBP, and DBP. Although the quartile 3 and quartile 4 OR values were lower than reference (quartile 1), the 95% CI showed a nonsignificant result, and the risk of depressive symptoms did not increase progressively with the quartiles of the CVAI index (*P* for trend = 0.199). Moreover, we found that CVAI was significantly correlated to depressive symptoms when analyzed as continuous variable (OR, 0.89; 95% CI, 0.79-0.98).  Figure 1(a) presents the dose-response curve of CVAI and the risk of depressive symptom. Our findings suggested a U-shaped relationship between CVAI and depressive symptoms, rather than a clear dose-response relationship (*P* = 0.673, *P* for nonlinear = 0.455). After excluding the top 2.5% and bottom 2.5% of CVAI values, the U-shaped relationship between CVAI and depressive symptoms remained unchanged (Figure 1(b)). This suggested that even after removing extreme values, the U-shaped pattern remained consistent. The risk of developing depressive symptoms continuously decreased as CVAI gradually increased. However, there was no significant association between them if CVAI exceeded 60.87.

### 3.3. Serial Multiple Mediation

**Figure 2:**
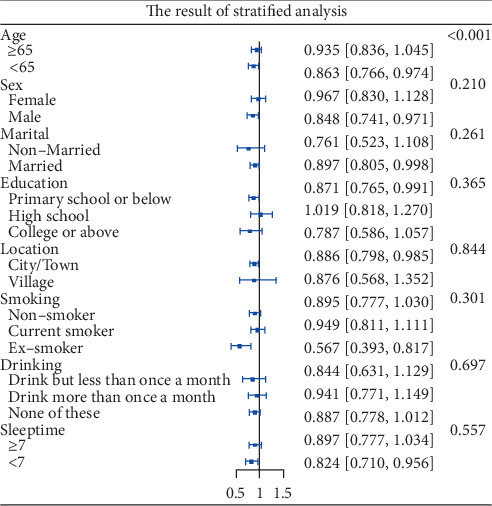
Path diagram of the serial mediating effects of sleep time and life satisfaction on the relationship between CVAI index and depressive symptoms.

Four statistically significant paths were determined using serial multiple model analysis (Figure 2). Age, gender, education level, location, marital status, smoking status, drinking status, sleep time, diabetes, SBP, and DBP were all examined as covariates. Results from the serial multiple mediation are shown in Table 3. Sleep time (a1b1; effect = −0.00, 95% CI: -0.001 to -0.000) and life satisfaction (a2b2; effect = −0.001, 95% CI: -0.001 to -0.000) presented mediating effects on the association between CVAI and depressive symptoms. The path of sleep time and life satisfaction (a1⁣^∗^d12⁣^∗^b2, effect = −0.000, 95% CI: -0.003 to 0.000) showed that there were statistically significant serial multiple mediator effects between CVAI and depressive symptoms. We proposed that a significant correlation between the CVAI index and depressive symptoms was mediated by a number of factors such as sleep time and life satisfaction (50.00% indirect effect/total effect), with the latter factor having a relatively significant influence.

### 3.4. Stratified Analysis

**Figure 3:**
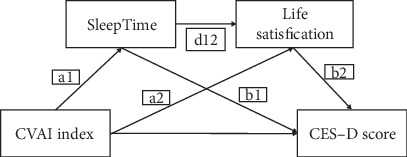
Forest plot of stratified analysis of the relationship between CVAI index and the risk of depressive symptoms. It showed that there were significant interactions between CAVI and age. OR: odds ratio; CI: confidence intervals.

The participants were grouped by characteristics to determine whether the CVAI index would influence the risk of depressive symptoms among the various subgroups. The influence of the CVAI index on the risk of depressive symptoms was not consistent among subgroups, according to the results. There was a substantial link between age and CVAI (*P* for interaction 0.001). Each IQR increase in CVAI was associated with 13.7% (95% CI: 0.026-0.234) decreased risk of depressive symptoms for participants aged < 65, but not for participants aged ≥ 65 (Figure 3).

## 4. Discussions

This study explored the association between CVAI and depressive symptoms based on the data from CHARLS in 2011 and 2015. To the best of our knowledge, this was the first study investigating such association in a large-scale population with restricted cubic spline analysis. In addition, we further investigated the mediating effect of sleep time and life satisfaction on the relationship between CVAI and depressive symptoms. We found that the participants with depressive symptoms were more likely to be female, nonmarried, less educated, living in city/town, less life satisfied, nondrinker, nonsmoker, less slept, and lower BMI. After adjusting for all covariates, CVAI levels were negatively associated with the risk of depressive symptoms. Restricted cubic spline regression showed a U-shaped relationship between CVAI and the risk of depressive symptoms. Stratified analysis showed that participants younger than 65 years would benefit more from CVAI index. Serial multiple mediation model indicated that approximately 50.00% (indirect effect/total effect) of the significant association between CVAI and depressive symptoms was mediated by factors such as sleep time and life satisfaction, with life satisfaction playing a comparatively significant role.

Although depression had been linked to genetic, physiological, and psychosocial factors, its pathogenesis was not yet entirely understood. Previous studies suggested a comorbidity between depressive symptoms and obesity and confirmed their common biological mechanisms, including inflammation, poor glycemic control, and dysregulation of the hypothalamic-pituitary-adrenal axis [21, 22]. However, there was a lack of high-quality prospective cohort studies, while cross-sectional studies could not conclude the cause-and-effect relationship between depressive symptoms and obesity. In addition, some clinical studies suggested controversial findings on the relationship between depressive symptoms and obesity when using BMI and waist circumference as indexes of obesity. A meta-analysis with 204,507 participants suggested a significant positive association between depressive symptoms and obesity (OR = 1.26; 95% CI; 1.17–1.36, *P* ≤ 0.001). But a cross-sectional study from South Korea came to the opposite conclusion: the higher the BMI, the lower the risk of depressive symptoms [23]. According to a UK study, neither depression nor obesity was linked to an increased incidence of MDE (major depressive episodes) [24]. Therefore, further research into the association between obesity and depressive symptoms was necessary.

A growing number of studies confirm that, compared to BMI and WC, abdominal obesity is a more reliable predictor for the risk of diseases such as cardiovascular and metabolic diseases. Similar findings have been concluded in studies related to depression. A cross-sectional study with 65,648 participants suggested that visceral fat distribution was a key mediator on the relationship between obesity and depression. However, a higher BMI was not independently correlated with depressive symptoms [25]. VAI is an important indicator in assessing visceral adiposity and is a reliable predictor for various metabolic diseases such as cardiometabolic disorders, diabetes, and nonalcoholic fatty liver disease [26, 27]. Few previous studies have examined the association between VAI and the risk of depression symptoms, and no studies examined their dose-response relationship. Lei et al. suggested that the clinical depression increased by 14% with each one unit increase in VAI (OR = 1.14, 95% CI: 1.04-1.25). A higher VAI score was associated with higher risk of developing depression [28]. Cho et al. suggested similar findings [29]. However, our study suggested a significant negative correlation between CVAI and the risk of depressive symptoms. These different results could be explained as follows: (1) different study populations: Lei's study included U.S. adults ≥ 18 years, and Cho's study included the general Korean population without age restriction, while this study included middle-aged and elderly Chinese ≥ 45 years; (2) different diagnostic criteria for depression: Lei used a PHQ-9 score ≥ 10 as the criterion for depression, and Cho used a total BDI score ≥ 16 as the criterion for having clinical depressive symptoms, while our study used the CES-D 10 scale to assess the severity of depressive symptoms; and (3) different confounders adjusted: Lei adjusted various factors such as gender, age, race, education level, marital status, diabetes, family income-to-poverty ratio, self-reported chronic diseases, WC, BMI, smoking status, dietary intake in a 24 h period, lipid indexes, vitamin D, glycohemoglobin, and fasting blood glucose; Cho adjusted factors including gender, age, BMI, total abdominal fat, hypertension, and diabetes mellitus, while this study adjusted factors including age, gender, education level, location, marital status, diabetes mellitus, smoking status, drinking status, blood pressure, and sleep time. Therefore, differences in research approaches, age of participants, and cultural backgrounds might provide possible explanation for these disparate findings.

In our study, we found that there was a U-shaped relationship between CVAI and the risk of depressive symptoms. It can be interpreted that an appropriate amount of visceral fat is associated with lower depressive symptoms. This finding is critical because it challenges the conventional belief that high visceral fat level is always harmful to health. Some previous epidemiological studies on elderly adults suggested findings that were in line with our study. Ho et al. [30] analyzed the cross-sectional data of 2604 community-dwelling Chinese ≥ 55 years and found that participants with moderate to high BMI had lower risk of depression compared to those with normal BMI. Palinkas et al. [31] suggested similar conclusions based on data from the U.S. population. “Jolly fat” hypothesis [32] and the dietary habit of obese population might help to explain these findings. Obesity implied an adequate and abundant source of food which, to some extent, might alleviate depressive symptoms [33]. In addition, obese people might tend to consume more carbohydrate-rich food, bringing pleasure and comfort. In traditional Chinese beliefs, obesity in middle-aged and elderly people is considered a symbol of health, strength, and good fortune [34]. The well-known expression “happy mind and fat body” originated in a Chinese classic that appeared about 2000 years ago. Overweight or obese male are more satisfied with their bodies [35], while long-lasting dieting and weight loss may contribute to a high incidence of depressive symptoms [36, 37]. And cognitive function may mediate the relationship between appropriate levels of visceral obesity and lower risk of depressive symptoms [38]. Visceral fat and estrogen levels were positively associated [39]. Estrogen modulates the expression of brain-derived neurotrophic factor and hippocampus synaptic plasticity, which enhances cognitive function [40]. The improvement of cognitive function may help to relieve depressive symptoms to a certain extent [41]. Therefore, an appropriate level of visceral fat may help to maintain a good mental state.

Most people retire at the age of 65, who are likely to develop depressive symptoms due to their social role changes and declined physical fitness [42]. Therefore, we conducted age-grouped stratified analysis to explore the correlation between CVAI and depressive symptoms. Results showed that there was significant interaction between CVAI and age (*p* for interaction < 0.001) and younger participants trended to benefit more from CVAI. First, elderly people commonly suffer from chronic pain, a contributor to daily life inconveniences, sense of shame, social isolation, and financial burden, which are major causes of depressive symptoms [43, 44]. Second, long-term medication of painkillers may bring side effects such as urinary retention, constipation, and cognitive impairment [45]. Compared to middle-aged people, elderly ones are more likely to develop various underlying comorbidities including cardiovascular and respiratory diseases [46, 47]. Therefore, we speculated that the negative effects of chronic pain and other underlying diseases may have weakened the impact of CVAI on the psychological status of elderly people.

In the serial multiple mediation model, we found that about 50.00% of the significant association between CVAI and depressive symptoms was mediated by life satisfaction and sleep time, with life satisfaction playing a comparatively major role. This could be interpreted as that compared to participants with high CVAI; those with low CVAI were more likely to be less satisfied with life, resulting in depressive symptoms. One possible explanation is that lower level of visceral fat implies dietary restriction and mandatory physical activity [48], which to some extent diminish people's sense of well-being and satisfaction. People with higher life satisfaction tend to feel happier, contributing to a lower risk of depressive symptoms [49]. Previous studies suggested that underweight was associated with decreased physical and mental health-related quality of life [50]. And we found that sleep time mediated the association between CVAI and depressive symptoms, which meant that visceral fat content might influence sleep time, leading to depressive symptoms. One possible explanation would be that high visceral fat content or obesity was closely linked to obstructive sleep apnea (OSA), which would seriously affect the duration and quality of sleep [51]. Sleep deprivation would contribute to the development of depression [52]. Therefore, guaranteed sleep time and quality might help lower the risk of obesity and depression [53]. Our findings suggested that improving life satisfaction and sleep time might alleviate depressive symptoms in middle-aged and elderly people with low body fat.

### 4.1. Strengths

First, it was a prospective cohort study covering a large and representative population sample from rural China, with reliable conclusion; second, we explored the mediating effect of sleep time and life satisfaction on the association between CVAI and depressive symptoms, which might provide a different perspective on the alleviation of depressive symptoms.

### 4.2. Limitations

First, the diagnosis of depression was based on the timing of self-reported depressive symptoms in the previous week, which might be subject to recall bias; second, due to limited data, we were unable to account for all potential confounders, such as dietary intakes and physical activities; third, the study population was middle-aged and elderly people, and it was still unknown whether the findings could be generalized to other age groups, such as adults under 45 years old or children and adolescents. Future cohort studies with larger samples and wider population coverage are needed to better elucidate the correlation between visceral adiposity and the risk of depressive symptoms. Finally, it should be noted that although CVAI is a useful index for assessing visceral fat accumulation in Asians, it does not directly measure the amount of visceral fat like computed tomography (CT) scans.

## 5. Conclusions

CVAI may be a valid indicator for identifying and predicting depression in middle-aged and elderly population. The risk of depressive symptoms decreases with the increase of CVAI, so appropriate visceral adiposity may help with depression prevention. Middle-aged and elderly people with lower visceral adiposity may need closer attention for early detection of depressive symptoms. Our study confirms that life satisfaction predominantly mediates the association between CVAI and depressive symptoms. Improving life satisfaction may help alleviate depressive symptoms in middle-aged and elderly Chinese with lower CVAI.

## Figures and Tables

**Table 1:** Baseline characteristic of the study population according to CAVI quartile.

	Total (*n* = 4149)	Q1 (*n* = 1038)	Q2 (*n* = 1037)	Q3 (*n* = 1037)	Q4 (*n* = 1037)	*P*
Age	57.80 ± 8.56	57.10 ± 8.49	56.80 ± 8.21	57.40 ± 8.48	59.90 ± 8.74	<0.001
Gender						<0.001
Female	1917 (46.204)	233 (22.447)	499 (48.12)	596 (57.473)	589 (56.798)	
Male	2232 (53.796)	805 (77.553)	538 (51.88)	441 (42.527)	448 (43.202)	
Marital						0.120
Nonmarried	329 (7.930)	75 (7.225)	74 (7.136)	80 (7.715)	100 (9.643)	
Married	3820 (92.070)	963 (92.775)	963 (92.864)	957 (92.285)	937 (90.357)	
Education						0.572
Primary school or below	2596 (62.569)	642 (61.85)	658 (63.452)	626 (60.366)	670 (64.609)	
High school	1009 (24.319)	255 (24.566)	246 (23.722)	266 (25.651)	242 (23.337)	
College or above	544 (13.112)	141 (13.584)	133 (12.825)	145 (13.983)	125 (12.054)	
Location						<0.001
City/town	3795 (91.468)	984 (94.798)	967 (93.250)	922 (88.910)	922 (88.910)	
Village	354 (8.532)	54 (5.202)	70 (6.750)	115 (11.090)	115 (11.090)	
Smoking						<0.001
Nonsmoker	2382 (57.411)	397 (38.247)	613 (59.113)	691 (66.635)	681 (65.670)	
Current smoker	1404 (33.839)	557 (53.661)	347 (33.462)	248 (23.915)	252 (24.301)	
Ex-smoker	363 (8.749)	84 (8.092)	77 (7.425)	98 (9.450)	104 (10.029)	
Drinking						<0.001
Drink but less than once a month	359 (8.653)	107 (10.308)	92 (8.872)	84 (8.100)	76 (7.329)	
Drink more than once a month	1214 (29.260)	444 (42.775)	288 (27.772)	239 (23.047)	243 (23.433)	
None of these	2576 (62.087)	487 (46.917)	657 (63.356)	714 (68.852)	718 (69.238)	
Diabetes						<0.001
No	3517 (84.767)	947 (91.233)	929 (89.585)	865 (83.414)	776 (74.831)	
Yes	632 (15.233)	91 (8.767)	108 (10.415)	172 (16.586)	261 (25.169)	
Life satisfaction	2.800 ± 0.623	2.870 ± 0.573	2.840 ± 0.627	2.780 ± 0.640	2.710 ± 0.639	<0.001
Sleep time	6.710 ± 1.630	6.650 ± 1.640	6.640 ± 1.630	6.780 ± 1.620	6.760 ± 1.620	0.089
SBP	126.333 (114.333, 140.667)	120.667 (110.417, 133)	122 (112, 136.667)	127.667 (116, 141.333)	134 (122.667, 147.333)	<0.001
DBP	75 (67.667, 83)	72.667 (65.083, 79.667)	73.333 (66, 81.333)	75.667 (68.667, 83.333)	78.333 (71, 86.667)	<0.001
BMI (kg/m^2^)	23.387 (21.23, 25.922)	20.587 (19.198, 22.012)	22.455 (21.016, 23.846)	24.261 (22.856, 25.834)	27.323 (25.429, 29.225)	<0.001
WC (cm)	85.00 (78.2, 92.0)	75.85 (71.4, 79.0)	82.3 (78.5, 85.0)	88.4 (84.8, 91.0)	97.0 (92.8, 101.0)	<0.001
Glucose (mg/dl)	102.42 (94.5, 113.04)	99.72 (92.52, 107.775)	100.62 (92.7, 109.26)	103.14 (95.4, 114.3)	107.82 (98.46, 122.04)	<0.001
TG (mg/dl)	105.315 (74.34, 155.76)	73.455 (57.525, 99.12)	92.04 (69.915, 129.21)	115.935 (86.73, 163.725)	161.07 (116.82, 240.72)	<0.001
HDL-C (mg/dl)	49.098 (40.206, 59.536)	59.923 (49.871, 71.038)	52.964 (45.232, 61.469)	46.392 (39.433, 53.737)	39.82 (33.248, 47.165)	<0.001
CVAI	93.856 (63.17, 126.375)	42.337 (23.438, 53.995)	79.241 (71.056, 86.277)	108.92 (101.528, 117.573)	148.055 (136.401, 164.508)	<0.001
Depression						0.646
No	3217 (77.537)	800 (77.071)	818 (78.881)	804 (77.531)	795 (76.663)	
Yes	932 (22.463)	238 (22.929)	219 (21.119)	233 (22.469)	242 (23.337)	

Note: *p* values were calculated from chi-square tests (categorical variables) or rank-sum tests (continuous variables without normal distribution) or ANOVA (continuous variables with normal distribution). Abbreviations: CVAI = Chinese visceral adiposity index; BMI = body mass index; WC = waist circumference; TG = triglyceride; HDL-C = high-density lipoprotein cholesterol; SBP = systolic blood pressure; DBP = diastolic blood pressure.

**Table 2:** Association between CVAI and the risk of depressive symptoms in the CHARLS.

	Model 1	*P*	Model 2	*P*	Model 3	*P*
CVAI per IQR	0.982 [0.897, 1.074]	0.691	0.893 [0.810, 0.986]	0.026	0.885 [0.798, 0.981]	0.020
Q1 (≤63.2)	Ref		Ref		Ref	
Q2 (≤93.9)	0.900 [0.730, 1.110]	0.320	0.760 [0.620, 0.950]	0.015	0.760 [0.610, 0.950]	0.015
Q3 (≤126.0)	0.970 [0.790, 1.200]	0.802	0.810 [0.650, 1.000]	0.053	0.810 [0.640, 1.010]	0.058
Q4 (≤263.0)	1.020 [0.830, 1.250]	0.826	0.840 [0.670, 1.050]	0.121	0.830 [0.660, 1.040]	0.106
*P* for trend	0.657		0.222		0.199	

Note: model 1 contained only independent variables; model 2 adjusted for age, gender, level of education, location, and marital status; model 3 adjusted further for smoking status, drinking status, sleep time, SBP, and DBP (model 3). Abbreviations: CVAI = Chinese visceral adiposity index; IQR = interquartile range.

**Table 3:** Indirect effects between CVAI and depression symptoms through different mediators.

	Estimate	S.E.	*z*	*P*	[Boot 95% CI]
Indirect_All	-0.001	0.000	-4.757	<0.001	[-0.002, -0.001]
Ind_X_M1_Y	0.000	0.000	-2.879	0.004	[-0.001, -0.000]
Ind_X_M2_Y	-0.001	0.000	-3.917	<0.001	[-0.001, -0.000]
Ind_X_M1_M2_Y	0.000	0.000	-1.929	0.054	[-0.000, -0.000]
Direct	-0.001	-0.001	-1.465	0.143	[-0.003, 0.000]
Total	-0.002	-0.001	-2.727	0.006	[-0.004, -0.001]

Note: number of bootstrap samples for bias corrected bootstrap confidence intervals: 10,000. CI = confidence interval; LL = lower level; UL = upper level; SE = standard error.

## Data Availability

Data were gathered from the China Health and Retirement Longitudinal Study (CHARLS), a publicly available database that could be accessed at http://charls.pku.edu.cn.
